# Pyk2 activates the NLRP3 inflammasome by directly phosphorylating ASC and contributes to inflammasome-dependent peritonitis

**DOI:** 10.1038/srep36214

**Published:** 2016-10-31

**Authors:** I-Che Chung, Chun-Nan OuYang, Sheng-Ning Yuan, Hsin-Pai Li, Jeng-Ting Chen, Hui-Ru Shieh, Yu-Jen Chen, David M. Ojcius, Ching-Liang Chu, Jau-Song Yu, Yu-Sun Chang, Lih-Chyang Chen

**Affiliations:** 1 Molecular Medicine Research Center, Chang Gung University, 333 Taoyuan, Taiwan; 2 Department of Microbiology and Immunology, Chang Gung University, 333 Taoyuan, Taiwan; 3 Graduate Institute of Biomedical Sciences, Chang Gung University, 333 Taoyuan, Taiwan; 4 Division of Hematology-Oncology, Chang Gung Memorial Hospital at Lin-Kuo, 333 Taoyuan, Taiwan; 5 Department of Medical Research Mackay Memorial Hospital, 251 New Taipei City, Taiwan; 6 Department of Radiation Oncology, Mackay Memorial Hospital, 251 New Taipei City, Taiwan; 7 Department of Biomedical Sciences, University of the Pacific Arthur A. Dugoni School of Dentistry, San Francisco, CA 94103, USA; 8 Graduate Institute of Immunology, College of Medicine, National Taiwan University, 100 Taipei, Taiwan; 9 Proteomics Core Laboratory, Chang Gung University, 333 Taoyuan, Taiwan; 10 Liver Research Center, Chang Gung Memorial Hospital at Lin-Kou, 333 Taoyuan, Taiwan; 11 Department of Otolaryngology, Chang Gung Memorial Hospital at Lin-Kuo, 333 Taoyuan, Taiwan; 12 Department of Medicine, Mackay Medical College, 252 New Taipei City, Taiwan

## Abstract

The inflammasome adaptor protein, ASC, contributes to both innate immune responses and inflammatory diseases via self-oligomerization, which leads to the activation of the protease, caspase-1. Here, we report that the cytosolic tyrosine kinases, FAK and Pyk2, are differentially involved in NLRP3 and AIM2 inflammasome activation. The inhibition of FAK and Pyk2 with RNA interference or chemical inhibitors dramatically abolished ASC oligomerization, caspase-1 activation, and IL-1β secretion in response to NLRP3 or AIM2 stimulation. Pyk2 is phosphorylated by the kinase Syk and relocalizes to the ASC specks upon NLRP3 inflammasome activation. Pyk2, but not FAK, could directly phosphorylate ASC at Tyr146, and only the phosphorylated ASC could participate in speck formation and trigger IL-1β secretion. Moreover, the clinical-trial-tested Pyk2/FAK dual inhibitor PF-562271 reduced monosodium urate-mediated peritonitis, a disease model used for studying the consequences of NLRP3 activation. Our results suggest that although Pyk2 and FAK are involved in inflammasome activation, only Pyk2 directly phosphorylates ASC and brings ASC into an oligomerization-competent state by allowing Tyr146 phosphorylation to participate ASC speck formation and subsequent NLRP3 inflammation.

The inflammasome is a cytoplasmic multiprotein complex composed of various pattern recognition receptors (PRRs), such as nod-like receptors (NLRs), AIM2, or RIG-I, along with the adaptor protein, apoptosis-associated speck-like protein containing CARD (ASC), and pro-caspase-1^1^. The formation of an inflammasome requires the oligomerization of ASC and the subsequent assembly of ASC specks. These specks recruit and activate the protease caspase-1^2^,^3^, which causes inflammation through the cleavage of pro-interleukin 1 beta (pro-IL-1β) or pro-interleukin 18 (pro-IL-18) to the mature proinflammatory cytokines, IL-1β and IL-18^1^. Among the numerous inflammasomes identified, the NLR family, pyrin domain-containing 3 (NLRP3) inflammasome is the best characterized to date. It is induced by pathogen-associated molecular patterns (PAMPs), microbial toxins (e.g., nigericin), and damage-associated molecular patterns [DAMPs; e.g., ATP and monosodium urate (MSU)]. The NLRP3 inflammasome has been shown to participate in development of cancer, as well as various inflammation-related diseases, including gout, diabetes and Alzheimer’s disease^1^.

Most PRRs involved in innate immunity use kinase-mediated protein phosphorylation to transduce damage signals into immunological effector responses^4^. Not surprisingly, a number of kinases [e.g., PKR^5^, AMPK^6^, Syk^7^,^8^,^9^, Lyn^10^, PI(3)K^11^, BTK^12^, and DAPK^13^] have been implicated in regulation of the NLRP3 inflammasome. However, their precise mechanisms of action have not been elucidated yet. Recently, Spalinger *et al*. has shown that phosphorylation of endogenous ASC is induced in bone marrow-derived dendritic cells by NLRP3 stimulator, MSU^14^. In addition, other groups found that phosphorylation of ASC at Tyr146 is critical for its oligomerization in response to NLRP3 stimulation, and that the phosphorylation and oligomerization of ASC requires the kinase activity of Syk. However, studies examining whether ASC is a Syk substrate have yielded contradictory results^7^,^8^,^15^. Moreover, the roles of other kinases involved in the direct phosphorylation of the NLRP3 inflammasome are largely unknown.

The focal adhesion kinase (FAK) family members, including proline-rich tyrosine kinase 2 (Pyk2) and FAK, act as cytoplasmic tyrosine kinases. Pyk2 and FAK are multifunctional proteins that primarily promote cell migration by controlling the disassembly of focal adhesions to extend the leading edge and retract the trailing edge. Integrin-ligand interactions activate Pyk2 and FAK in part by triggering their autophosphorylation at Tyr402 and Tyr397, respectively^16^,^17^. This, in turn, controls the rearrangement of the actin cytoskeleton by regulating Rho and Rac signals^16^. Pyk2 and FAK are also activated by T cell receptor signals, and contribute to the development and activation of T cells^17^. In cancer, FAK is a therapeutic target, as it can promote cell proliferation by increasing cyclin D1 expression and inducing the epithelial-mesenchymal transition by down-regulating the cell surface expression of E-cadherin^16^.

We recently showed that inflammasomes recruit neutrophils in nasopharyngeal carcinoma (NPC), and that elevated expression levels of inflammasome components were correlated with better patient survival^18^. We further demonstrated that inflammasome activation is regulated by AMPK via modulation of the inflammasome- and microtubule-associated protein, end-binding protein 1 (EB1)^19^,^20^. However, it still remains unclear how protein kinases regulate the key adaptor protein ASC in the inflammasomes, or the role that cytoskeleton-associated kinases may play.

Here, we report for the first time that the phosphorylation of Pyk2 at Tyr402 is increased following the treatment of nigericin, an activator of NLRP3 inflammasome, and that p-Pyk2 co-localizes with ASC in ASC specks. Furthermore, we show that Pyk2 can bind ASC and directly phosphorylate it at Tyr146, and that only phosphorylated ASC can form oligomers and trigger IL-1β secretion. These results show that Pyk2 can regulate inflammation by directly targeting the key adaptor protein, ASC.

## Results

### FAK family kinases are putative kinases for the phosphorylation of ASC Tyr146

Tyr146 in human ASC is equivalent to Tyr144 in mouse ASC; the phosphorylation of this residue is controlled by Syk and is critical for NLRP3-dependent ASC speck formation and IL-1β secretion^7^,^8^. However, it is not clear whether ASC is directly phosphorylated by Syk^15^. Here, we utilized an online kinase prediction algorithm (PhosphoNET; http://www.phosphonet.ca/) to search for kinases that could potentially catalyze Tyr146 phosphorylation of ASC. Supplementary Table S1 presents the top 50 protein kinases that are likely to phosphorylate ASC Tyr146 based on the Kinase Predictor V2 Score. The FAK family kinases, Pyk2 and FAK, ranked among the top candidates, and were thus considered to be the most likely candidates for the phosphorylation of ASC at Tyr146.

### Pyk2 and FAK are differentially required for inflammasome activation, as assessed by NLRP3- and AIM2-mediated IL-1β secretion

**Figure 1:**
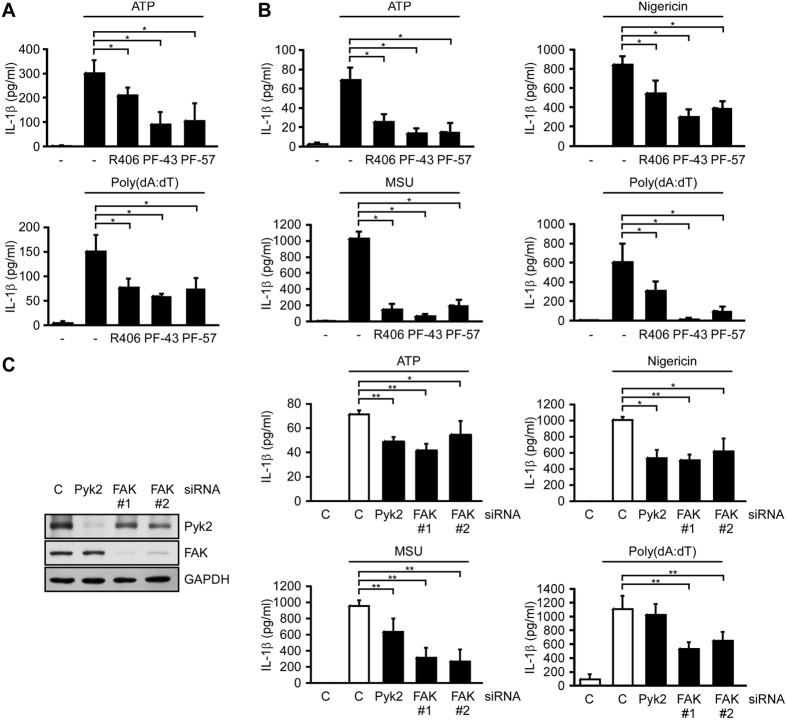
Pyk2 and FAK are essential for NLRP3-mediated IL-1β secretion. (**A**) ELISA was used to assess the IL-1β level in culture supernatants of human monocyte-derived macrophages stimulated with ATP or poly(dA:dT) in the presence or absence of the kinase inhibitors, R406, PF-431396 (PF-43), or PF-573228 (PF-57). (**B**) ELISA of IL-1β in the culture supernatant of PMA-differentiated THP-1 cells stimulated with ATP, MSU, nigericin, or poly(dA:dT) in the presence or absence of the indicated kinase inhibitors. (**C**) ELISA of IL-1β in the supernatant of PMA-differentiated THP-1 cells treated with Pyk2-, FAK-, or negative control (**C**) siRNA, followed by stimulation with ATP, MSU, nigericin, or poly(dA:dT). The knockdown efficiencies of Pyk2 and FAK are shown in the left panel. Two independent FAK siRNA are indicated as FAK#1 and FAK#2. The western blot is a representative of three independent experiments. Symbols: *P < 0.05; and **P < 0.01. All results are presented as the mean ± SD of three independent experiments, and were analyzed with the Student’s t test.

To examine the role of Pyk2 and FAK in NLRP3- and AIM2-dependent secretion of IL-1β, we treated human monocyte-derived macrophages with the Pyk2/FAK dual inhibitor, PF-431396, the FAK-specific inhibitor, PF-573228, or the Syk inhibitor, R406 (positive control, as Syk was previously shown to be critical for ASC phosphorylation and IL-1β secretion^7^,^8^). We found that IL-1β secretion in response to ATP and poly(dA:dT) (which stimulate NLRP3 and AIM2 respectively) was significantly inhibited by all three inhibitors (Fig. 1A). To study the mechanisms involved in Pyk2- and FAK-mediated inflammasome activation, we performed further experiments in PMA-differentiated THP-1 cells. Our results revealed that PF-431396 and PF-573228 significantly inhibited IL-1β secretion and pyroptosis, as measured by the release of lactate dehydrogenase (LDH) by THP-1 cells stimulated with the NLRP3 stimulators, ATP, the ionophore nigericin, and monosodium urate (MSU) crystals, and AIM2 stimulator, poly(dA:dT) (Fig. 1B and Supplementary Fig. S1A), but not affected pro-IL-1β expression (Supplementary Fig. S2A). To differentiate the roles of Pyk2 and FAK in inflammasome regulation, we examined IL-1β secretion, pro-IL-1β expression, and LDH release in response to NLRP3 and AIM2 activation in THP-1 cells in which Pyk2 and FAK had been knocked down by small interfering RNA (siRNA). We found that individual depletion of Pyk2 or FAK significantly inhibited NLRP3-mediated IL-1β secretion (Fig. 1C) and LDH release (Supplementary Fig. S1B), rather than affected pro-IL-1β expression (Supplementary Fig. S2B). In addition, the depletion of FAK, but not Pyk2, significantly inhibited AIM2-mediated IL-1β secretion and LDH release.

### Phosphorylation of Pyk2 is the downstream of Syk signaling

**Figure 2:**
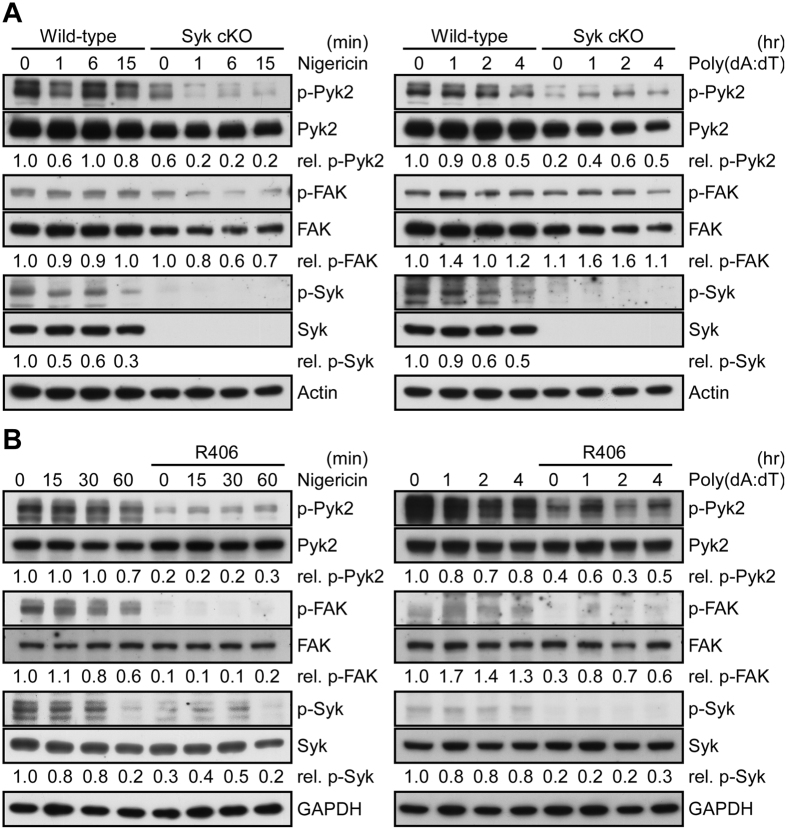
Phosphorylation of Pyk2 is the downstream of Syk signaling. (**A**) Immunoblot analysis of p-Pyk2, p-FAK, and p-Syk in PECs from *Syk*^flox/flox^/LysM-Cre(−) (wild-type), and *Syk*^flox/flox^/LysM-Cre(+) (Syk cKO) mice. Those cells were stimulated with nigericin (left panel) and poly(dA:dT) (right panel) for a period as indicated. The western blot is a representative of three independent experiments. The ratio of p-Pyk2, p-FAK, and p-Syk in the blot were calculated by normalization with total Pyk2, FAK, Syk, respectively, using the values from the unstimulated control cells as 1.0. (**B**) Immunoblot analysis of p-Pyk2, p-FAK, and p-Syk in PMA-differentiated THP-1 cells that have been pretreated with R406 for 1 h, following stimulation with nigericin (left panel) and poly(dA:dT) (right panel) for a period as indicated. The western blot is a representative of two independent experiments. The ratio of p-Pyk2, p-FAK, and p-Syk in the blot were calculated by normalization with total Pyk2, FAK, Syk, respectively, using the values from the unstimulated control cells as 1.0.

We then clarified whether Pyk2 and FAK are Syk downstream signaling activated by NLRP3 and AIM2 stimuli. As shown in Fig. 2A, levels of p-Syk, p-Pyk2, and p-FAK were not affected upon the stimulation of nigericin and poly(dA:dT) in macrophages from wild-type mice. The knockout of *Syk* significantly decreases p-Pyk, but not p-FAK. We also assessed the effect of Syk inhibitor, R406 in THP-1 cells, p-Pyk2 and p-FAK were inhibited by R406, although both were not affected by nigericin and poly(dA:dT) (Fig. 2B). Taken together, this suggested that Pyk2 acted downstream of Syk signaling in macrophages in the absence of inflammasome stimulation, which is also required for the activation of NLRP3 inflammasome.

### Pyk2 and FAK are involved in caspase-1 activation and the formation of ASC specks

**Figure 3:**
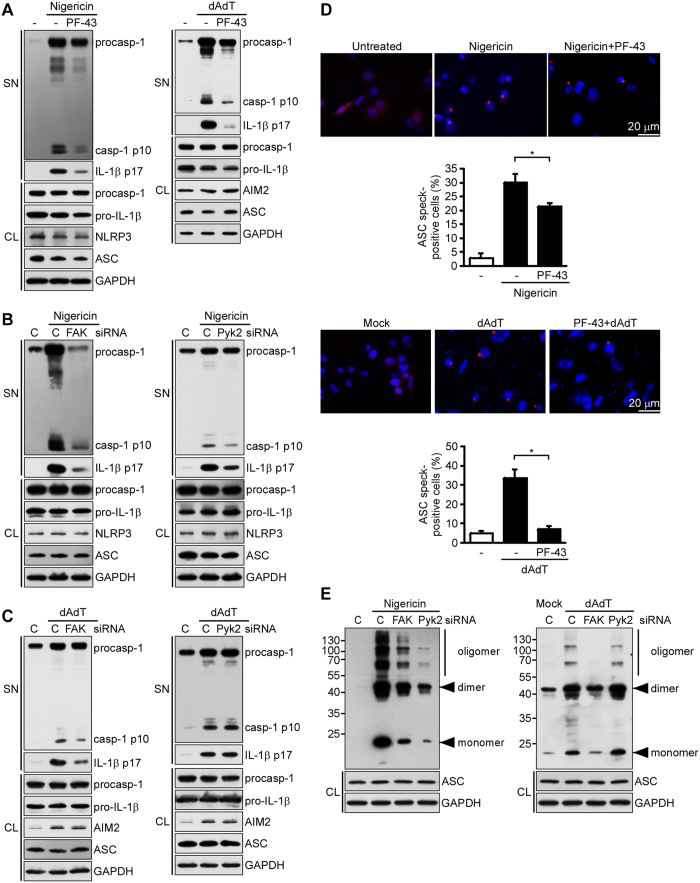
Pyk2 and FAK are essential for NLRP3 inflammasome. (**A**) Immunoblot analysis of NLRP3 and AIM2 inflammasome molecules in culture supernatants (SN) and cell lysates (CL) of THP-1-derived macrophages that had been treated with PF-431396 (PF-43) and then stimulated with nigericin (left panel) and poly(dA:dT) (right panel). (**B**) Immunoblot analysis of NLRP3 inflammasome molecules in cell supernatants and cell lysates of THP-1-derived macrophages treated with Pyk2- (left panel), FAK- (right panel), or negative control (**C**) siRNA, and then stimulated with nigericin. (**C**) Immunoblot analysis of AIM2 inflammasome molecules in cell supernatants and cell lysates of THP-1-derived macrophages treated with Pyk2- (left panel), FAK- (right panel), or negative control (**C**) siRNA, and then stimulated with poly(dA:dT). (**D**) Quantification of ASC specks in ASC-mCherry-expressing THP-1 cells treated with PF-431396 and then stimulated with nigericin (top panel) and poly(dA:dT) (bottom panel). The percentage of cells with ASC specks, as determined by an IN Cell Analyzer. (**E**) Analysis of ASC oligomerization in THP-1-derived macrophages treated with FAK-, Pyk2-, or negative control (**C**) siRNA, and then stimulated with nigericin. Expression level of ASC protein in cell lysates (CL) was measured (bottom panel). The western blot is a representative of three independent experiments. Abbreviations: Casp-1 p10, active caspase-1 subunit; procasp-1, p45 precursor of caspase-1; IL-1β p17, secreted mature IL-1β; and pro-IL-1β, p31 precursor of IL-1β. Data are representative of at least three independent experiments.

NLRP3 and AIM2 recruits caspase-1 through ASC, allowing activated caspase-1 to cleave pro-IL-1β to mature IL-1β^1^. Here, we assessed the involvement of Pyk2 and FAK in nigericin- and poly(dA:dT)-induced activation of caspase-1 (assessed by the presence of p10) and IL-1β (assessed by production of the cleaved form, p17) in THP-1 cells. We found that activation was dramatically blocked by the Pyk2/FAK dual inhibitor, PF-431396 (Fig. 3A). To further distinguish which kinases are required for NLRP3 and AIM2 activation, we transfected THP-1 cells with siRNA targeting Pyk2 or FAK. Our data demonstrated that depletion of Pyk2 or FAK significantly reduced nigericin-induced caspase-1 activation (Fig. 3B). However, depletion of FAK, but not Pyk2, significantly reduced poly(dA:dT)-induced caspase-1 activation (Fig. 3C). In addition, the level of mature IL-1β p17 was consistent with the status of caspase-1 activation (as assessed by the level of caspase-1 p10) (Fig. 3B,C). These results suggested that both Pyk2 and FAK are involved caspase-1 activation and IL-1β secretion in nigericin-stimulated NLRP3 inflammasomes.

In these inflammasomes, ASC must be oligomerized to recruit pro-caspase-1 prior the activation of caspase-1. To investigate whether Pyk2 and FAK contribute to ASC speck formation in THP-1 cells upon the activation of NLRP3 or AIM2 inflammasomes, we microscopically observed and counted the formation of mCherry ASC specks in the presence and absence of the Pyk2/FAK dual inhibitor, PF-431396. Our results revealed that PF-431396 reduced nigericin- or poly(dA:dT)-induced ASC speck formation (Fig. 3D). Moreover, depletion of Pyk2 or FAK in THP-1 cells greatly decreased nigericin-induced ASC oligomerization rather than affected ASC expression level (Fig. 3E). However, depletion of FAK, but not Pyk2, reduced poly(dA:dT)-induced ASC oligomerization (Fig. 3E). These results demonstrate that Pyk2 and FAK are required for activation of the ASC-caspase-1-IL-1β axis in nigericin-stimulated NLRP3 inflammasomes.

### Pyk2 interacts with ASC and colocalizes with ASC specks upon NLRP3 inflammasome activation

**Figure 4:**
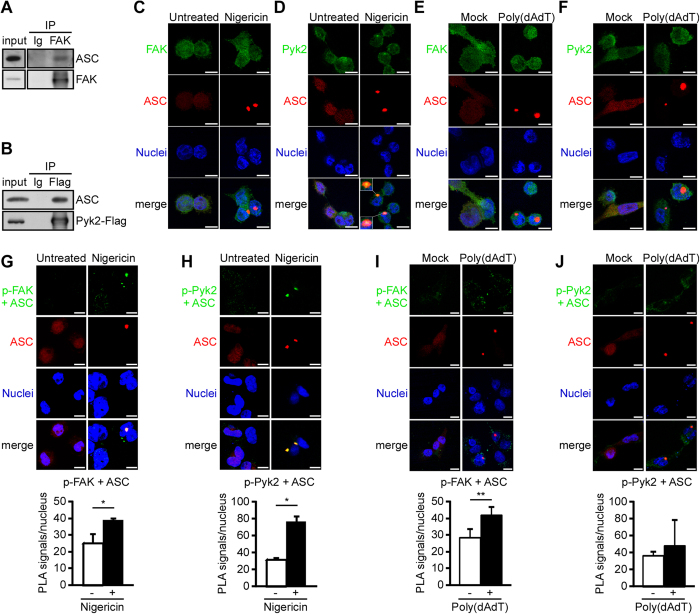
Interaction of Pyk2 and FAK with ASC. (**A**) Immunoblot analysis of FAK and ASC in immunoprecipitants prepared with anti-FAK and the corresponding normal rabbit IgG from extracts of HEK293T cells obtained at 48 h after transfection of a vector encoding ASC-Flag. The western blot is a representative of three independent experiments. (**B**) Immunoblot analysis of Pyk2-Flag and ASC in immunoprecipitants prepared with anti-Flag matrix and the corresponding normal mouse IgG, from HEK293T cell extracts obtained 48 h after co-transfection of vectors encoding Pyk2-Flag and ASC. (**C–F**) The localization of FAK and Pyk2 in ASC-mCherry-expressing THP-1 cells stimulated for 1 h with nigericin (**C**,**D**) and for 4 h with poly(dAdT) (**E**,**F**) was visualized by immunostaining with an anti-FAK or anti-Pyk2 antibody (green). ASC is shown in red, while nuclei are blue. Scale bars, 10 μm. (**G–J**) *In situ* PLA of PMA-differentiated THP-1 cells stimulated for 1 h with nigericin (**G**,**H**) and for 4 h with poly(dAdT) (**I**,**J**). (**G**,**I**) Complexes of phosphorylated FAK with ASC (p-FAK + ASC, green). (**H**,**J**) Complexes of phosphorylated Pyk2 with ASC (p-Pyk2 + ASC, green). ASC is shown in red, while nuclei are blue. The results were quantified using an IN Cell Analyzer, and are presented relative to the value obtained from unstimulated control cells. Scale bars, 10 μm.

To examine whether Pyk2 and FAK could be involved in directly phosphorylating ASC at Tyr146 (Table S1) and whether they are both required for the formation of ASC specks (Fig. 3D,E), we used co-immunoprecipitation, immunofluorescence staining, and proximity ligation assays (PLA) to examine whether Pyk2 and FAK interact with ASC. As shown in Fig. 4A,B, ASC proteins could be co-immunoprecipitated from ASC-overexpressing HEK293T cells when immunoprecipitation was performed using endogenous FAK (as assessed using a FAK-specific antibody) (Fig. 4A) or overexpressed Pyk2-Flag (as assessed using a Flag tag-specific antibody) (Fig. 4B), but not immunoglobulin G (negative control). This suggested that Pyk2 and FAK have the ability to form a complex with ASC. For further examining this possibility, we detected the co-localization of Pyk2 and FAK with ASC in the absence or presence of inflammasome stimulation (Fig. 4C–F). As shown in Fig. 4D, a part of Pyk2 colocalized with the ASC specks upon nigericin stimulation, although the distribution of major Pyk2 and FAK was not affected upon stimulation. PLA was used to confirm whether ASC interacts with p-Pyk2 and p-FAK *in situ*. The signals of ASC-p-FAK complex and ASC-p-Pyk2 complex were detectable in ASC-mCherry-expressing THP-1 cells with or without stimulation (Fig. 4G–J). Following nigericin stimulation, the signals of ASC-p-Pyk2 complex were stronger and larger, and also colocalized with the ASC specks (red in Fig. 4H). In contrast, the effect could not be detected in cells treated with poly(dAdT). Taken together, these results clearly revealed that Pyk2 and FAK interact with ASC in THP-1 cells in the cytoplasm in the absence of stimulation. In addition, the activation of NLRP3 by nigericin enhances the interaction of Pyk2 with ASC and triggers the co-localization of Pyk2 with ASC in the ASC specks.

### Pyk2 directly phosphorylates ASC at Tyr146 to activate the NLRP3 inflammasome

**Figure 5:**
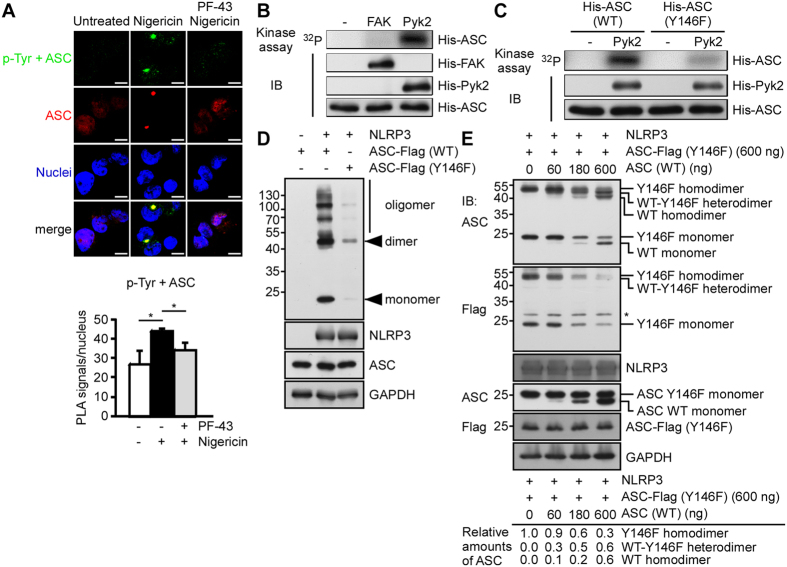
ASC Tyr146 is phosphorylated by Pyk2, and this is essential for ASC oligomerization. (**A**,**B**) *In situ* PLA of phosphorylated tyrosine-ASC complexes in PMA-differentiated THP-1 cells stimulated for 1 h by nigericin in the presence or absence of PF-431396. (**A**) Complexes of phosphorylated tyrosine with ASC (p-Tyr + ASC; green). ASC is shown in red, while nuclei are blue. The results were quantified with an IN Cell Analyzer, and are presented relative to the value obtained from unstimulated control cells. Scale bars, 10 μm. (**B**) An *in vitro* kinase assay of FAK and Pyk2 was performed by incubating recombinant His-ASC with His-FAK or His-Pyk2, as indicated. The protein amounts were assessed by immunoblotting with anti-FAK, anti-Pyk2, and anti-ASC antibodies. (**C**) An *in vitro* kinase assay of Pyk2 was performed by incubating recombinant His-Pyk2 with wild-type or mutant (Y146F) His-ASC. The protein amounts were assessed by immunoblotting with anti-Pyk2 and anti-ASC antibodies. (**D**) Analysis of ASC oligomerization in reconstituted HEK293T cells 48 h after co-transfection of empty vector or Flag-NLRP3-encoding vectors plus wild-type or mutant (Y146F) ASC-Flag-encoding vectors. The western blot is a representative of three independent experiments. (**E**) Analysis of ASC oligomerization in reconstituted HEK293T cells 48 h after co-transfection of vectors encoding Flag-NLRP3 or mutant (Y146F) ASC-Flag along with increasing amount of vector encoding wild-type ASC, and immunoblotting was performed as indicated using anti-ASC (top panel) and anti-Flag (middle panel) antibodies. The western blot is a representative of three independent experiments. Relative amounts of mutant ASC (Y146F) homodimers, wild-type/mutant ASC heterodimers, and wild-type ASC homodimers from this representative blot are shown at the bottom panel. Symbol: *non-specific signal.

Phosphorylation of ASC Tyr146, which is required for the formation of ASC specks, is controlled by Syk and Jnk via an unknown pathway^7^,^15^. Our present data suggested that ASC forms the complex with p-FAK and p-Pyk2 (Fig. 4G–J) and the expression of p-Pyk2 is the downstream of Syk signaling (Fig. 2). To investigate whether the formation of ASC specks is regulated by the Pyk2- and/or FAK-mediated phosphorylation of ASC, we visualized tyrosine-phosphorylation signal within ASC complexes (green). This signal colocalized with the ASC specks in ASC-mCherry-expressing THP-1 cells, as assessed by *in situ* PLA (Fig. 5A). Nigericin treatment induced a strong tyrosine-phosphorylation signal within ASC complexes, and this induction was blocked by the pretreatment with the Pyk2/FAK inhibitor, PF-431396 (Fig. 5A).

Next, we examined whether Pyk2 and FAK could phosphorylate ASC directly by using purified recombinant His-tagged ASC as the substrate for *in vitro* Pyk2 and FAK kinase assays. The results revealed that ASC could be phosphorylated by His-tagged Pyk2, but not by His-tagged FAK (Fig. 5B). To confirm that Tyr146 of ASC was the key amino acid residue phosphorylated by Pyk2, we generated a mutant His-ASC (Y146F), and used it as the substrate for a kinase assay. The mutant ASC (Y146F) showed lower ^32^P incorporation compared to wild-type His-ASC, indicating that Tyr146 is the major phosphorylation site on ASC, and its phosphorylation is Pyk2-dependent (Fig. 5C). To elucidate the contribution of this specific phosphorylation to NLRP3 inflammasome function, we examined the ability of wild-type or mutant ASC (Y146F) to form ASC oligomers in HEK293T cells subjected to stimulation of NLRP3 expression. Mutant ASC (Y146F) assembled into almost no ASC oligomers in response to stimulation of NLRP3 expression, whereas wild-type ASC showed notable oligomerization under the same conditions (Fig. 5D). This indicates that phosphorylation of ASC Tyr146 is critical for formation of the NLRP3 inflammasome. To further test whether wild-type ASC could function as a nucleation factor to recruit either wild-type or mutant ASC to form dimers to perpetuate ASC oligomerization, we introduced various amounts (60, 180, and 600 ng) of wild-type ASC into HEK293T cells along with 600 ng of mutant ASC (Y146F). As shown in Fig. 5E, top panel, the expression of wild-type ASC yielded similar levels of wild-type ASC homodimers and wild-type/mutant ASC heterodimers, indicating that wild-type ASC could act as a nucleation factor to equally utilize wild-type and mutant ASC to perpetuate ASC oligomerization. Unexpectedly, increasing expression of wild-type ASC competitively reduced the formation of mutant ASC homodimers (Fig. 5E, middle panel), indicating that mutant ASC is less efficient than wild-type ASC with regards to ASC dimer formation. The data, taken together, indicate that while wild-type ASC equally utilizes wild-type and mutant ASC to perpetuate ASC oligomerization, mutant ASC is less efficient than wild-type ASC to utilize mutant ASC to perpetuate ASC oligomerization. Recently, caspase recruitment domain (CARD) of ASC is shown to be involved in the cross-linking of ASC filaments and mediates the assembly of dense ASC speck and caspase-1 activation^21^,^22^. Since ASC Tyr146 is at CARD domain, we examined whether the inhibition of Pyk2 signaling would affect the cross-linking of ASC. As shown in the Supplementary Fig. S3, the treatment of PF-431396 induced more long filaments of ASC specks and resulted in a significant increase in diameter of ASC specks in ASC-mCherry-expressing THP-1 cells in response to NLRP3 activation by nigericin. Our results thus show for the first time that Tyr146 of ASC is phosphorylated directly by Pyk2, in a step that is critical for the NLRP3-mediated ASC speck formation.

### Effect of the Pyk2/FAK dual inhibitor on MSU-induced peritonitis

**Figure 6:**
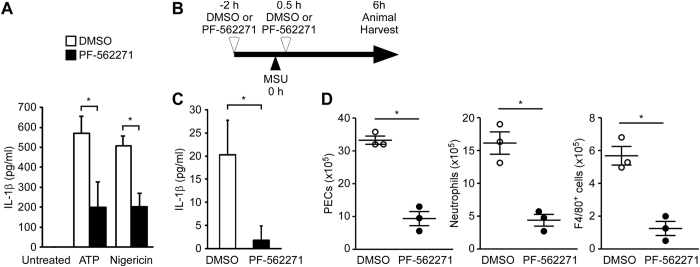
Involvement of Pyk2 and FAK in MSU-induced inflammatory responses *in vivo*. (**A**) ELISA of IL-1β in the supernatant of mouse BMDMs primed for 4 h with LPS and then stimulated with ATP (for 4 h) or nigericin (for 1 h) in the presence or absence of PF-562271. All results are presented as the mean ± SD of three independent experiments, and were analyzed using the Student’s t test. (**B**) Schematic presentation of MSU-induced peritonitis in mice. (**C**) ELISA of IL-1β in the peritonea of mice treated for 6 h with intraperitoneal injection of MSU in the presence or absence of PF-562271. (**D**) Absolute number of peritoneal exudate cells (PECs), CD11b^+^/Ly6G^+^/F4/80^−^ neutrophils, and CD11b^+^/F4/80^+^ monocytes-macrophages in the peritonea of mice treated for 6 h with intraperitoneal injection of MSU in the presence or absence of PF-562271. Each symbol represents an individual mouse (n = 3 per group); small horizontal lines indicate the mean (±s.d.). Symbol: *P < 0.05.

To determine the biological significance of Pyk2 and FAK in NLRP3 inflammasome activation *in vivo*, we analyzed an indicator of inflammation (the recruitment of inflammatory cells into the peritoneal cavity) following intraperitoneal injection of mice with MSU^7^. Mouse bone marrow-derived macrophages (BMDMs) were obtained and treated with ATP or nigericin in the presence and absence of the clinical trial-tested Pyk2/FAK dual inhibitor, PF-562271. Consistent with our findings in human monocyte-derived macrophages and THP-1 cells (Fig. 1A,B), the IL-1β secretion induced by ATP or nigericin was significantly blocked by pretreatment of BMDMs with PF-562271 (Fig. 6A). Next, we analyzed the inhibition effect of PF-562271 on MSU-induced peritonitis (Fig. 6B). *In vivo*, MSU induced the production of IL-1β and the recruitment of cells (e.g., Gr-1 + F4/80− neutrophils and F4/80 + monocytes and macrophages) to the peritoneal cavity. However, pretreatment with PF-562271 significantly reduced the amounts of IL-1β and the numbers of recruited cells, compared to the DMSO control (Fig. 6C,D). These results suggest that the PF-562271-induced blockade of Pyk2 and FAK signaling reduces IL-1β production and the recruitment of inflammatory cells to the peritoneal cavity, thus alleviating the inflammatory symptoms in this *in vivo* model.

## Discussion

**Figure 7:**
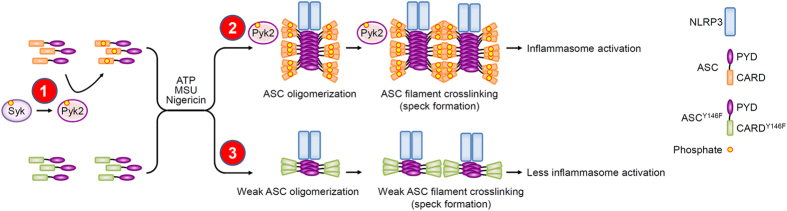
Model for Pyk2-dependent NLRP3 inflammasome activation. Based on our present results, we propose that: (1) in the absence of NLRP3 stimulus, p-Pyk2 (Tyr402) acts downstream of Syk signaling to phosphorylate CARD of ASC at Tyr146 in the cytoplasm and that bring ASC into an oligomerization-competent state; (2) in the presence of NLRP3 stimulus, NLRP3 oligomers recruit ASC molecules, trigger ASC oligomerization, and then form dense ASC speck by the cross-linking of ASC filament. The CARD of ASC is critical for the cross-linking and mediates the assembly of dense ASC speck, caspase-1 activation, and consequently activates NLRP3 inflammasome; and (3) expression of ASC Y146 mutant significantly abolishes ASC phosphorylation, ASC oligomerization, the cross-linking of ASC filaments to form dense ASC speck, and inflammasome formation.

The FAK family kinases, Pyk2 and FAK, regulate cell migration by disassembling focal adhesions to extend the leading edge and retract the trailing edge of cells^16^. This requires dynamic rearrangement of the actin cytoskeleton, which is achieved by the association of Pyk2 and FAK with GRAF, paxillin, and other proteins, which in turn activate signaling by Rho and Rac^16^. In innate immunity, Pyk2 and FAK can contribute to immune cell migration (e.g., macrophages) to the infection site^23^. Upon infection, proinflammatory cytokines that serve as alarm signals (e.g., IL-1β) are secreted from resident macrophages, and recruit phagocytes to infected tissues for clearance of pathogens. Here, we demonstrate that both Pyk2 and FAK are involved in NLRP3 activation. We further examined the mechanism whereby Pyk2 regulates NLRP3 inflammasome activation, as summarized in the proposed model (Fig. 7). We show that p-Pyk2 (Tyr402) is expressed in macrophages in the absence of inflammasome stimulus, and that Pyk2 phosphorylate Tyr146 at ASC CARD by directly binding ASC and that brings ASC into an oligomerization-competent state. Upon NLRP3 stimulation, phosphorylated ASC molecules are recruited to form ASC filament by ASC oligomerization, and then form dense ASC speck by the cross-linking of ASC filament, and consequently activates NLRP3 inflammasome. This enables that Pyk2 brings ASC into an oligomerization-competent state to form dense speck, and regulate NLRP3-mediated IL-1β secretion.

Protein phosphorylation-mediated signal transduction is mechanistically important for the regulation of many biological processes, including inflammation. The involvement of kinases such as PKR^5^, AMPK^6^, Syk^7^,^8^,^9^, Lyn^10^, PI(3)K^11^, BTK^12^, and DAPK^13^ in the activation of the NLRP3 inflammasome have been reported, yet their precise mechanisms of action are not known. Recently, phosphorylation of endogenous ASC was observed in bone marrow-derived dendritic cells upon MSU-mediated NLRP3 activation^14^. Syk-mediated phosphorylation of ASC Tyr146 was shown to be critical for ASC oligomerization in response to NLRP3 stimuli in macrophages^7^,^8^. However, it is not clear how ASC phosphorylation mediates the oligomerization of ASC and whether other tyrosine kinases are involved. Here, we show that ASC is specifically phosphorylated by Pyk2 but not by FAK (Fig. 5B). The Pyk2-mediated phosphorylation of the ASC (Y146F) mutant was significantly reduced compared with wild-type ASC (Fig. 5C), indicating that Tyr146 of ASC is the major phosphorylation site for Pyk2. The ASC (Y146F) mutant still evoked a weak ^32^P signal in the kinase assay, suggesting that there are additional phosphorylation sites for Pyk2 on ASC. Hara *et al*. have suggested other putative phosphorylation sites of ASC, S58 and T151-153^7^. While these amino acids could be involved in both ASC speck formation and IL-1β-inducing ability, they look unlikely to be directly phosphorylated by Pyk2, due to that Pyk2 is a tyrosine kinase. In fact, using PhosphoNET to predict the possible consensus phosphorylation sites for Pyk2 on ASC, we identified three additional candidate tyrosines: Y60, Y137, and Y187. Moreover, we show that the mutant ASC (Y146F) can efficiently form heterodimers with wild-type ASC but does not efficiently form mutant homodimers in the presence of wild-type ASC (Fig. 5E). This strongly suggests that ASC phosphorylation is critical for the recruitment of ASC molecules during the oligomerization of ASC in specks. Consistent with this notion, the p-Pyk2-ASC complex is present in the cytoplasm in the absence of inflammasome stimulus, and then colocalized with ASC specks upon NLRP3 stimulation (Fig. 4H). Thus, it seems reasonable to hypothesize that Pyk2 phosphorylates ASC in the cytoplasm, and that brings ASC into oligomerization-competent status for ASC oligomerization, cross-linking of ASC filaments, and speck formation upon NLRP3 activation (Fig. 7).

Our results suggest that both FAK and Pyk2 are required to activate NLRP3 inflammasomes and caspase-1, and that this occurs via ASC oligomerization (Fig. 3B,E). However, FAK, unlike Pyk2, does not phosphorylate ASC. We speculate that FAK may regulate NLRP3 inflammasomes through the phosphorylation of targets other than ASC and/or by acting as an adaptor protein. Recent reports by our group and Misawa *et al*. showed that microtubule-associated protein EB1 and microtubule polymerization are required for the assembly of NLRP3 and AIM2 inflammasomes^19^,^24^,^25^. EB1 and AIM2 inflammasomes were shown to colocalize with γ-tubulin in the perinuclear region (i.e., the microtubule organizing center; MTOC) in macrophages subjected to poly(dA:dT) stimulation^19^,^25^. Pyk2 and FAK also localize with the MTOC via an association with paxillin, and thereby control microtubule polymerization and MTOC polarization^16^,^17^. This suggests that Pyk2 and FAK may regulate microtubule polymerization and MTOC polarization in response to NLRP3 stimuli. Furthermore, EB1 is involved in microtubule stabilization, which contributes to cell migration^26^,^27^,^28^. Given these findings, it is not surprising that Pyk2, FAK, and EB1 can promote both migration of inflammatory cells and inflammasome activation, thus regulating early stages of an immune response in a synchronous manner.

Pyk2 is reported to critically contribute (via a yet unknown mechanism) to IL-1β secretion in macrophages stimulated by the HIV-1 envelope glycoprotein, gp120^29^. Consistent with a previous report^7^, we found that the secretion of IL-1β from activated NLRP3 inflammasomes was reduced in macrophages treated with the Syk inhibitor, R406 (Fig. 1A,B). Syk is known to be required for Pyk2 phosphorylation^30^,^31^, which is involved in IFN-α-induced Jak activation and STAT1-dependent gene expression in macrophages^31^. However, no previous studies have examined whether Pyk2 is involved in Syk-mediated regulation of inflammasomes. Here, we show for the first time that Pyk2 is one of the key kinase responsible for phosphorylating ASC, which in turn allows the NLRP3 inflammasome activation. In addition, Pyk2 phosphorylation and NLRP3-mediated IL-1β secretion were suppressed by the Syk inhibitor, R406 (Figs 1A,B and 2B). Our results collectively indicate that Pyk2 is likely downstream of Syk in macrophages. Combining this with the previous findings, we propose that the Syk-Pyk2 signaling pathway is probably involved in both the interferon response and NLRP3 inflammasome activation.

Aberrant activation of the NLRP3 inflammasome contributes to the progression of many chronic diseases, including gout, Alzheimer’s disease, type II diabetes, atherosclerosis, and cancer^1^,^18^. Since inhibition of inflammasome formation may significantly reduce the damage caused by inflammation, inflammasomes are often regarded as a potential therapeutic target for these diseases^32^. Here, we show that phosphorylation of the inflammasome component, ASC, is a prerequisite for ASC oligomerization and inflammasome activation, and that this effect is controlled by the novel inflammasome regulators, Pyk2 and FAK. PF-562271, which is a potent ATP-competitive dual inhibitor of both Pyk2 and FAK, has completed phase I clinical trial (NCT00666926) and is considered to be a promising drug for patients with solid tumors^32^. In a mouse model, we found that PF-562271 can inhibit NLRP3-mediated IL-1β secretion and reduce MSU-induced peritonitis (Fig. 6). Our results collectively suggest that Pyk2 and FAK may serve as therapeutic targets for inflammation-related diseases. Further investigation of the various inflammasome components should help to unravel the mechanisms whereby kinases contribute to inflammasome activation, and identify additional mediators through which inflammasome activity may be controlled.

## Materials and Methods

### Reagents, antibodies, and plasmids

PMA, ATP, nigericin, PF-431396, 4′,6-diamidino-2-phenylindole (DAPI), and the anti-Flag M2 antibody were purchased from Sigma. R406, PF-562271, and Z-VAD-FMK were purchased from Selleckchem. Anti-Pyk2¸ anti-Syk, anti-p-Pyk2, anti-p-FAK, anti-p-Syk, and anti-GAPDH were purchased from Cell Signaling. Anti-FAK, anti-ASC, anti-caspase-1, and anti-IL-1β were purchased from Santa Cruz. Anti-Ly6G-PE and anti-CD11b-APC were purchased from BD Bioscience. MSU, PF-573228, anti-NLRP3, anti-mCherry, anti-F4/80-FITC, and Alexa Fluor 488–conjugated goat anti-rabbit IgG were purchased from InvivoGen, Tocris Bioscience, AdipoGen, Abcam, eBioscience, and Invitrogen, respectively. Plasmids encoding His-tagged wild-type and mutant (Y146F) ASC were generated by ligation of amplified DNA fragments to *Nde*I/*XhoI*-treated pET15b (Novagen, EMD Millipore, Darmstadt, Germany). The His-tagged mutant ASC (Y146F) was constructed using a QuikChange Site-Directed Mutagenesis kit (Stratagene) with forward primer 5′- GACGG ATGAG CAGTT CCAGG CAGTG CGGGC -3′ and reverse primer 5′- GCCCG CACTG CCTGG AACTG CTCAT CCGTC -3′. The mutant sequences are underlined. pENTER-Pyk2-Flag was purchased from ViGene Biosciences. The expression vectors for ASC-Flag and ASC were constructed using the pLKO_AS2 vector (RNAi Core, Taiwan), and that for Flag-NLRP3 was constructed using the pFLAG-CMV2 vector (Sigma).

### Animal experiments

Mouse experiments were performed under the ethical approval by the Institutional Animal Care and User Committee of Chang-Gung University, and the methods were carried out in accordance with the approved guidelines. Female wild-type C57BL/6J, *Syk*^flox/flox^, and LysM-Cre mice were maintained under specific pathogen–free conditions and used at 6–9 weeks of age. For MSU-induced peritonitis, mice were intraperitoneally treated with dimethyl sulfoxide or PF-562271 (25 mg per kg body weight) at 2 h before and 30 minutes after challenge with MSU (1 mg). At 6 h after MSU treatment, peritoneal cells were collected. The number of total cells was determined by trypan blue staining. Neutrophils (CD11b^+^/Ly6G^+^) and macrophages (CD11b^+^/F4/80^+^) were subjected to antibody staining and analyzed with a FACSCalibur (Becton Dickinson).

### Tissue specimens and cell culture

Peripheral blood mononuclear cells were obtained from healthy donors using Histopaque density gradient centrifugation (GE Healthcare, Waukesha, WI). Informed consent was obtained from all volunteers prior to donation. Ethical approval for this study was obtained from Joint Institutional Review Board of the Mackay Memorial Hospital, and the methods were carried out in accordance with the approved guidelines. Human monocyte-derived macrophages were generated from human peripheral blood monocytes and cultured in α-MEM medium supplemented with 10% FCS and GM-CSF (PeproTech) for 7 days, as described previously^33^. Peritoneal exudate cells (PECs) of mice were obtained 3 days after an i.p. injection of 1 ml of 3% thioglycollate medium (Sigma) and cultured in RPMI 1640 medium supplemented with 10% FCS overnight at 37 °C. After incubation, the adherent PECs were used for this study. Mouse bone marrow-derived macrophages (BMDMs) were generated from bone marrow cells, which were collected from the tibias and femurs of C57BL/6J mice by flushing with cold PBS using a 25-G needle. Those cells were cultured in DMEM medium supplemented with 10% FCS and 10 ng/ml M-CSF (PeproTech) for 8 days. The THP-1 (human leukemia monocytic) cell line was purchased from Biosource Collection and Research Center (Taiwan) and maintained in RPMI as described previously^19^. The ASC-mCherry-expressing THP-1 cells were kindly provided by Dr. M. Z. Lai (Institute of Molecular Biology, Academia Sinica, Taipei, Taiwan)^20^. For macrophage differentiation, THP-1 cells were stimulated with 200 nM PMA for 16 h. For inflammasome stimulation, the cells were treated with 10 μM nigericin for 1 h, 5 mM ATP for 4 h, or 200 μg/ml MSU for 4 h. For signaling and caspase inhibition, R406 (3 μM), PF-431396 (10 μM), PF-573228 (10 μM), PF-562271 (10 μM), or Z-VAD-FMK (20 μM) were applied for 1 h prior to inflammasome stimulation.

### RNA interference

The dsRNA duplexes were purchased from Dharmacon and transfected into cells using Lipofectamine 2000 (Invitrogen), as previously described^18^. For efficient knockdown, the cells were incubated for two days. The oligonucleotide sequences of FAK#1 and FAK#2 were 5′-GGGCA UCAUU CAGAA GAUA-3′ and 5′-UAGUA CAGCU CUUGC AUAU-3′, respectively. The *SMART*pool reagent used to target Pyk2 included four 21-bp RNA duplexes: 5′-GGAUC AUCAU GGAAU UGUA -3′, 5′-UCAGU GACGU UUAUC AGAU-3′, 5′-GAAGA UGUGG UCCUG AAUC-3′, and 5′-GAGGA AUGCU CGCUA CCGA-3′.

### Immunoblot analysis

Cells were lysed in RIPA buffer (50 mM Tris-Cl, pH 7.5, 150 mM NaCl, 10 mM MgCl_2_, 1 mM EDTA, 1% Igepal CA-630) with a protease inhibitor cocktail (4.76 μg/ml leupeptin, 3.25 μg/ml aprotinin, 0.69 μg/ml pepstatin and 1 mM phenylmethylsulfonyl fluoride) on ice for 30 min. For assessment of IL-1β secretion and caspase-1 activation, culture supernatants were collected, mixed with a 1/10 volume of 100% (wt/vol) trichloroacetic acid, and incubated for 10 min at 4 °C. The precipitated protein samples were resolved by SDS/PAGE and transferred to PVDF membranes (Millipore). The membranes were incubated with the indicated primary antibodies, and then with an HRP-conjugated secondary antibody. The immunoreactive bands were detected using an enhanced chemiluminescence system (Amersham Pharmacia Biotech, AB, Uppsala, Sweden). Immunoblot images were quantified with ImageJ software.

### Immunoprecipitation

Cells were lysed in RIPA buffer with a protease inhibitor cocktail, and cell extracts (1 mg) were immunoprecipitated with anti-FAK or anti-FLAG M2 antibodies (1 μg) for 24 h. The corresponding rabbit and mouse IgGs (Millipore) were used as the control antibodies. The bound samples were precipitated with PureProteome Protein G Magnetic Beads (Millipore) for 1 h at 4 °C, and the immunoprecipitated products were collected for Western blot analysis.

### ASC oligomerization assay

THP-1 cells were lysed in buffer A (20 mM HEPES-KOH, pH 7.5, 10 mM KCl, 1.5 mM MgCl_2_, 1 mM EDTA, 1 mM EGTA, and 320 mM sucrose) supplemented with a protease inhibitor cocktail. The nuclei and unlysed cells were removed by centrifugation and 5 μm Ultrafree-CL centrifugal filters (Millipore). The filtrate was diluted with an equal volume of CHAPS buffer (20 mM HEPES-KOH, pH 7.5, 5 mM MgCl_2_, 0.5 mM EGTA, and 0.1% CHAPS) supplemented with a protease inhibitor cocktail, and centrifuged to obtain insoluble pelleted fraction. The pellets were resuspended in CHAPS buffer and then subjected to cross-linking using disuccinimidyl suberate (DSS; 2 mM) for 30 min. The protein samples were resolved by 12% SDS-PAGE, and the level of ASC was analyzed. For the reconstitution of NLRP3 inflammasomes, HEK293T cells were transfected with pCMV-Flag-NLRP3, pLKO_AS2-ASC-Flag, and pLKO_AS2-ASC (Y146F)-Flag using Lipofectamine 2000. They were then incubated for 48 h and analyzed as described for THP-1 cells. ASC speck formation was analyzed in ASC-mCherry-expressing THP-1 cells. ASC speck images were acquired under fluorescence microscopy (Olympus). For quantification, the ASC speck was counted with an IN Cell Analyzer (GE Healthcare) and normalized with respect to the number of nuclei, which were stained with DAPI.

### *In situ* proximity ligation assay (PLA)

The cells were washed and allowed to react with the following pairs of proximity probes (Olink Bioscience): rabbit anti-ASC and mouse anti-phosphotyrosine, rabbit anti-p-FAK and mouse anti-mCherry, and anti-p-Pyk2 and mouse anti-mCherry. *In situ* PLA was performed using the Duolink Proximity Ligation *in situ* reagent kit (Olink, Uppsala, Sweden), according to the manufacturer’s instructions. The results were visualized under confocal microscopy using a Zeiss LSM510 META laser scanning microscope (Carl Zeiss, Germany) with a 63 × 1.32 or 100 × 1.32 NA oil immersion objective. Quantification was performed with an IN Cell Analyzer (GE Healthcare).

### Immunofluorescence staining

The ASC-mCherry-expressing THP-1 cells were washed and allowed to react with the anti-FAK and anti-Pyk2 antibodies and sequentially incubated with an Alexa Fluor 488–conjugated secondary antibody. Nuclei were stained with DAPI. The results were visualized under confocal microscopy using a Zeiss LSM510 META laser scanning microscope (Carl Zeiss, Germany) with a 63 × 1.32 or 100 × 1.32 NA oil immersion objective.

### IL-1β ELISA

Cell culture supernatants were assayed for human IL-1β (eBioscience) and mouse IL-1β (eBioscience).

### *In vitro* kinase assay

For kinase assays, 200 ng of recombinant active kinase [FAK (393–698 aa; Sigma, F7680) or Pyk2 (360–690 aa; Sigma, K3768)] were incubated with purified ASC proteins (wild-type and mutant) and 30 μl of kinase buffer containing 60 mM HEPES, pH 7.5, 10 mM MgCl_2_, 5 mM MnCl_2_, 0.3 mM Na_3_VO_4_, 1.25 mM DTT, 0.2 mM ATP and 10 μCi of [γP^32^] ATP (Perkin Elmer Life Sciences, Waltham, MA). The kinase reactions were performed for 30 min at room temperature (RT) with shaking and stopped by the addition of SDS sample buffer, and the samples were heated at 95 °C for 5 min. The proteins were resolved by 12.5% SDS-PAGE, and phosphorylated proteins were detected by autoradiography.

### Purification of His-tagged fusion proteins

*Escherichia coli* BL21(DE3) cells were transformed with pET15b, grown to mid- exponential phase (OD 600, 0.5) at 37 °C, induced with 0.5 mM isopropylthiogalactoside (IPTG) for 6 h at room temperature (RT) and lysed by sonication in ice-cold 1x phosphate-buffered saline (PBS) supplemented with a protease inhibitor cocktail. The His-tagged fusion proteins were purified from bacterial lysates by incubation with a Ni Sepharose 6 Fast Flow column (GE Healthcare, Piscataway, NJ, USA) in accordance with the manufacturer’s instruction.

### Statistical analysis

All statistical analyses were performed using the SPSS 13.0 statistical software package. Data from *in vitro* experiments and mouse model were analyzed with the Student’s *t* test. Differences were considered significant at *P* < 0.05.

## Additional Information

**How to cite this article**: Chung, I.-C. *et al*. Pyk2 activates the NLRP3 inflammasome by directly phosphorylating ASC and contributes to inflammasome-dependent peritonitis. *Sci. Rep*. **6**, 36214; doi: 10.1038/srep36214 (2016).

**Publisher’s note**: Springer Nature remains neutral with regard to jurisdictional claims in published maps and institutional affiliations.

## Supplementary Material

Supplementary Information
