# Supplementary material for: Pyk2 activates the NLRP3 inflammasome by directly phosphorylating ASC and contributes to inflammasome-dependent peritonitis
Source: Sci Rep. 2016 Oct 31;6:36214. doi: 10.1038/srep36214 (PMC5087076; doi:10.1038/srep36214)
Supplement: Supplementary Information [file srep36214-s1.pdf]

Supplementary information

Pyk2 activates the NLRP3 inflammasome by directly phosphorylating ASC and contributes to inflammasome-dependent peritonitis

I-Che Chung[1], Chun-Nan OuYang[1], Sheng-Ning Yuan[1], Hsin-Pai Li[2,3,4], Jeng-Ting Chen[1], Hui-Ru Shieh[5], Yu-Jen Chen[5,6], David M. Ojcius[7], Ching-Liang Chu[8], Jau-Song Yu[3,9,10], Yu-Sun Chang[1,3,11], Lih-Chyang Chen[12,*]

[1]Molecular Medicine Research Center, Chang Gung University, 333 Taoyuan, Taiwan
[2]Department of Microbiology and Immunology, Chang Gung University, 333 Taoyuan, Taiwan
[3]Graduate Institute of Biomedical Sciences, Chang Gung University, 333 Taoyuan, Taiwan
[4]Division of Hematology-Oncology, Chang Gung Memorial Hospital at Lin-Kuo, 333 Taoyuan, Taiwan
[5]Department of Medical Research Mackay Memorial Hospital, 251 New Taipei City, Taiwan
[6]Department of Radiation Oncology, Mackay Memorial Hospital, 251 New Taipei City, Taiwan
[7]Department of Biomedical Sciences, University of the Pacific Arthur A. Dugoni School of Dentistry, San Francisco, CA 94103, USA
[8]Graduate Institute of Immunology, College of Medicine, National Taiwan University, 100 Taipei, Taiwan
[9]Proteomics Core Laboratory, Chang Gung University, 333 Taoyuan, Taiwan
[10]Liver Research Center, Chang Gung Memorial Hospital at Lin-Kou, 333 Taoyuan, Taiwan
[11]Department of Otolaryngology, Chang Gung Memorial Hospital at Lin-Kuo, 333 Taoyuan, Taiwan
[12]Department of Medicine, Mackay Medical College, 252 New Taipei City, Taiwan

[*]Corresponding author: Lih-Chyang Chen, Department of Medicine, Mackay Medical College, 252 New Taipei City, Taiwan. Tel: 886-2-26360303 ext 1225, email: lihchyang@mmc.edu.tw

Supplementary materials and methods

Lactate dehydrogenase (LDH) release assay

LDH levels were measured in the supernatant using the LDH Cytotoxicity detection kit (TaKaRa Clontech). The assays were performed according to the manufacturer's instructions, and the red formazan product was measured at 490 nm using a SpectraMax M2 microplate reader (Molecular Devices). Measurements were performed at least in triplicate. Percent LDH release was calculated as follows: [(LDH sample)-(LDH negative control)]/[(LDH positive control)-(LDH negative control)]. LDH negative control for spontaneous LDH release was measured from untreated cells. LDH positive control for maximum LDH release was measured in cells lysed with 2% Triton-X-100.

Measurements of speck sizes

ASC speck images were randomly acquired under confocal microscopy using a Zeiss LSM510 META laser scanning microscope (Carl Zeiss, Germany) with a 63×1.32 NA oil immersion objective. Nuclei were stained with DAPI. The largest diameters of individual specks were measured.

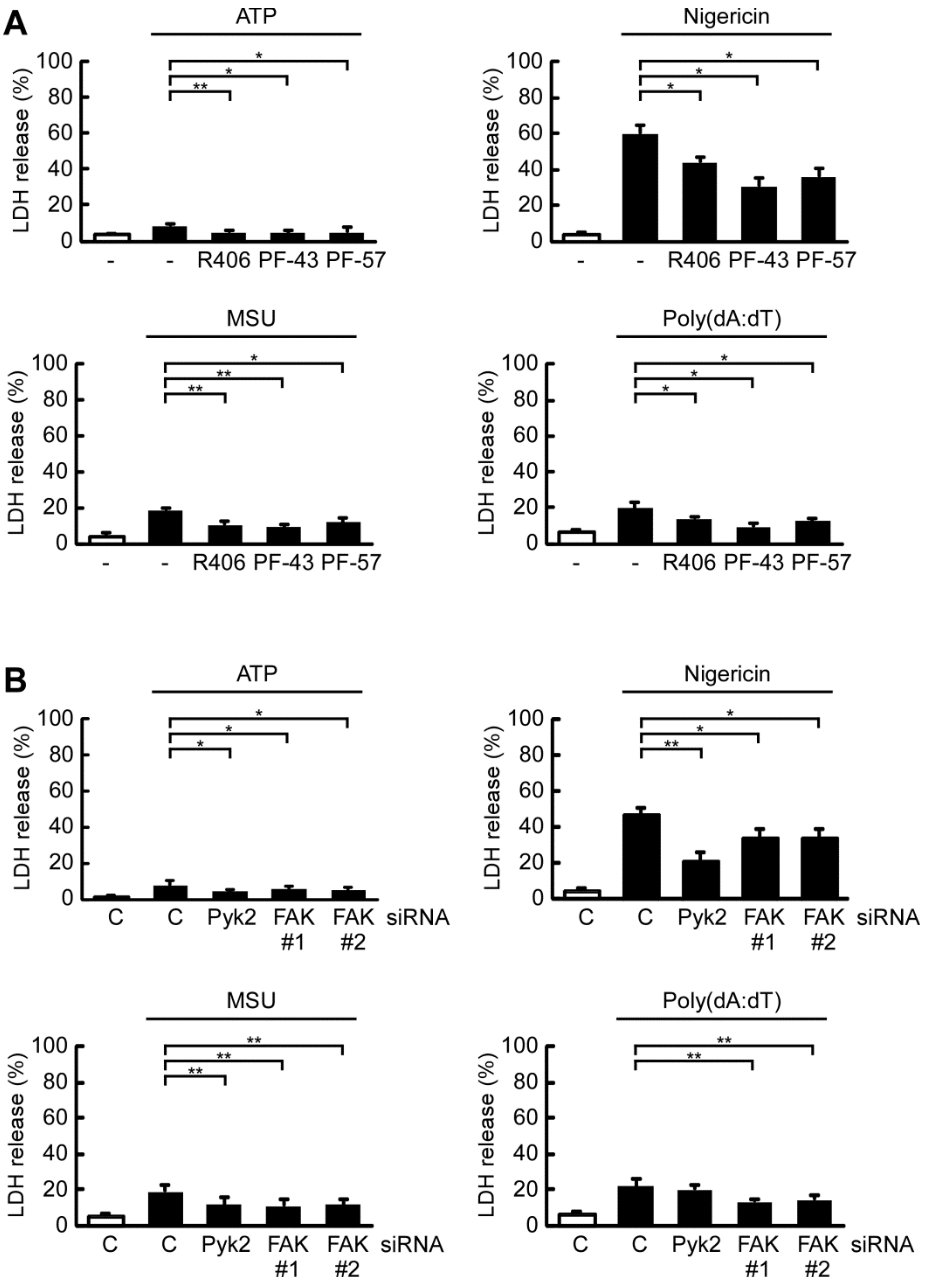

Supplementary Figure S1. Release of LDH upon NLRP3 and AIM2 inflammasome activation. (**A**) Activity assay of LDH in the culture supernatant of PMA-differentiated THP-1 cells stimulated with ATP, MSU, nigericin, or poly(dA:dT) in the presence or absence of the indicated kinase inhibitors. (**B**) Activity assay of LDH in the supernatant of PMA-differentiated THP-1 cells treated with Pyk2-, FAK-, or negative control (C) siRNA, followed by stimulation with ATP, MSU, nigericin, or poly(dA:dT). Two independent FAK siRNA are indicated as FAK#1 and FAK#2. Symbols: *, $P < 0.05$; and **, $P < 0.01$. All results are presented as the mean ± SD of three independent experiments, and were analyzed with the Student's t test.

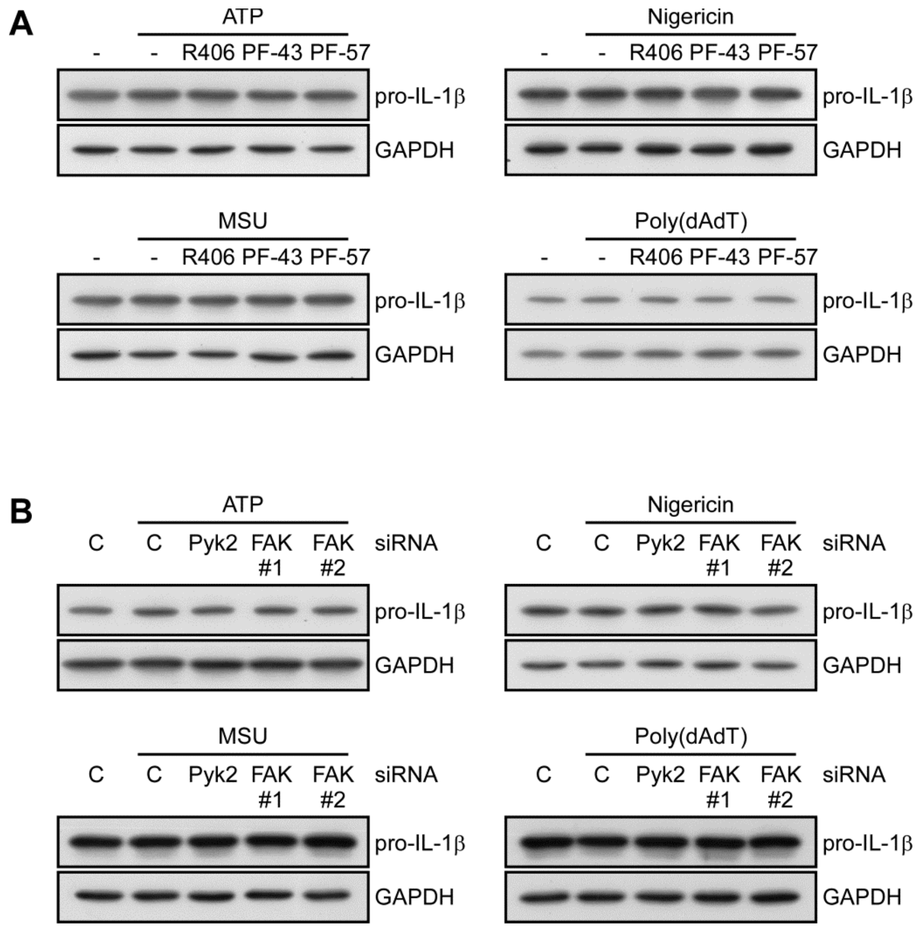

Supplementary Figure S2. Expression of pro-IL-1β upon NLRP3 and AIM2 inflammasome activation. (**A**) Western blotting of pro-IL-1β in the cell lysate of PMA-differentiated THP-1 cells stimulated with ATP, MSU, nigericin, or poly(dA:dT) in the presence or absence of the indicated kinase inhibitors. (**B**) Western blotting of pro-IL-1β in the cell lysate of PMA-differentiated THP-1 cells treated with Pyk2-, FAK-, or negative control (C) siRNA, followed by stimulation with ATP, MSU, nigericin, or poly(dA:dT). Two independent FAK siRNA are indicated as FAK#1 and FAK#2. The western blot is a representative of three independent experiments.

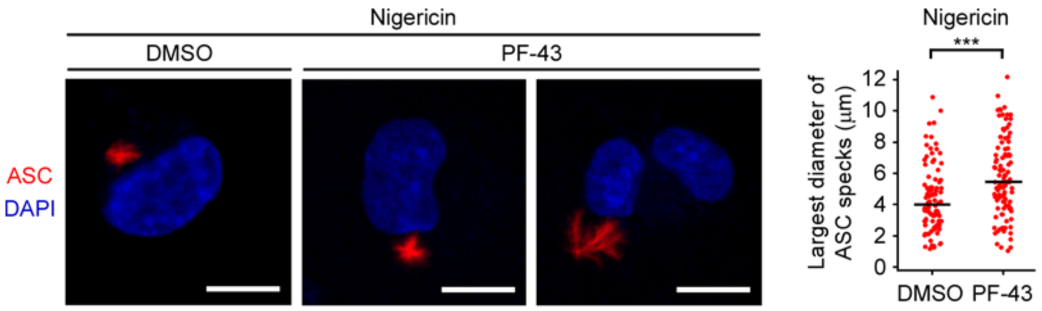

Supplementary Figure S3. Inhibition of condensed ASC specks by PF-431396. Representative images of ASC-mCherry-expressing THP-1 cells stimulated with nigericin in the presence or absence of PF-431396 (PF-43) for 1 h. Measurement of the ASC speck diameter was shown in right panel. The numbers of specks measured were 100 and 103 and the median speck size as indicated. Nuclei were stained with DAPI. Scale bars, 10 mm. ****P*<0.001 (one-way analysis of variance).

Table S1. Prediction of kinase-specific phosphorylation at ASC Tyr146

Protein kinase match	Human Kinase Short Name	Human UniProt. ID	Kinase Predictor V2 Score
Kinase 1:	PYK2 (PTK2B)	Q14289	463
Kinase 2:	FAK	Q05397	463
Kinase 3:	BRK	Q13882	457
Kinase 4:	MET (HGF Receptor)	P08581	457
Kinase 5:	ZAP70	P43403	455
Kinase 6:	CTK (MATK)	P42679	448
Kinase 7:	AXL	P30530	442
Kinase 8:	FER	P16591	442
Kinase 9:	FYN	P06241	440
Kinase 10:	SYK	P43405	438
Kinase 11:	KIT	P10721	434
Kinase 12:	MER (MERTK)	Q12866	434
Kinase 13:	YES1	P07947	434
Kinase 14:	FGFR1	P11362	432
Kinase 15:	SRC	P12931	428
Kinase 16:	CSK	P41240	427
Kinase 17:	HCK	P08631	426
Kinase 18:	ERBB3	P21860	424
Kinase 19:	TYRO3	Q06418	421
Kinase 20:	ERBB2	P04626	421
Kinase 21:	PDGFRA	P16234	420
Kinase 22:	TEC	P42680	418
Kinase 23:	FGFR4	P22455	417
Kinase 24:	ERBB4	Q15303	417
Kinase 25:	ABL	P00519	414
Kinase 26:	FGFR2	P21802	413
Kinase 27:	FGFR3	P22607	413
Kinase 28:	ARG (ABL2)	P42684	413
Kinase 29:	FRK	P42685	412
Kinase 30:	FES	P07332	412
Kinase 31:	FGR	P09769	409
Kinase 32:	FLT1	P17948	407
Kinase 33:	LYN	P07948	406
Kinase 34:	FMS (CSF1R)	P07333	405
Kinase 35:	RON	Q04912	405
Kinase 36:	EGFR	P00533	405
Kinase 37:	PDGFRB	P09619	405

Kinase 38:	FLT3	P36888	404
Kinase 39:	TXK	P42681	403
Kinase 40:	SRM (SRMS)	Q9H3Y6	399
Kinase 41:	BMX	P51813	398
Kinase 42:	RET	P07949	398
Kinase 43:	LCK	P06239	397
Kinase 44:	BLK	P51451	395
Kinase 45:	ALK	Q9UM73	394
Kinase 46:	Flt4 (VEGFR3)	P35918	394
Kinase 47:	ITK	Q08881	391
Kinase 48:	LTK	P29376	385
Kinase 49:	KDR	P35968	375
Kinase 50:	TIE2 (TEK)	Q02763	363

The top 50 human protein kinases that are likely to phosphorylate ASC Tyr146 as determined with PhosphoNET (http://www.phosphonet.ca/) based on Kinase Predictor V2 Score. The scores are provided by using kinase substrate prediction algorithm developed at Kinexus.
